# Comparative analysis of quantitative phosphoproteomics between two tilapias (*Oreochromis niloticus* and *Oreochromis aureus*) under low-temperature stress

**DOI:** 10.7717/peerj.15599

**Published:** 2023-07-10

**Authors:** Changgeng Yang, Hua Fan, Liya Ge, Qian Ma, Ming Jiang, Hua Wen

**Affiliations:** 1 Life Science & Technology School, Lingnan Normal University, Zhanjiang, China; 2 College of Fisheries, Guangdong Ocean University, Zhanjiang, China; 3 Fish Nutrition and Feed Division, Yangtze River Fisheries Research Institute, Chinese Academy of Fishery Sciences, Wuhan, China

**Keywords:** Tilapia, Phosphorylation, Omics, Hypothermy

## Abstract

As an important farmed fish, tilapia has poor tolerance to low-temperatures. At the same time, different tilapia strains have apparent differences in low-temperature tolerance. In this study, using the iTRAQ method, the phosphorylated proteomics of two tilapia strains (*Oreochromis niloticus* and* Oreochromis aureus*) with different tolerances to low-temperature stress were quantitatively and comparatively analyzed, to clarify the physiological mechanism of tilapia’s response to low-temperature stress. Through the GO and IPR analyses of differentially phosphorylated proteins, a number of similarities in physiological activities and regulatory effects were found between the two tilapias in response to low-temperature stress. Many differentially phosphorylated proteins are mainly involved in lipid metabolism, cell proliferation and apoptosis. However, the difference in endurance of low temperature of these two tilapias might be related to the differences in categories, expression and modification level of genetic products which were involved in the aforementioned physiological processes. And meanwhile, the enrichment results of KEGG showed the changes of multiple immune-related and growth-related phosphorylated proteins in the cytokine-cytokine receptor interaction pathway in *O. aureus* are more prominent. Furthermore, the significantly enriched pathway of carbohydrate digestion and absorption in *O. niloticus* may indicate that low-temperature stress exerts a more severe impact on energy metabolism. The relative results would help elucidating the molecular mechanism by which tilapia responds to low-temperature stress, and developing culture of tilapia species.

## Introduction

Tilapia (*Oreochromis spp.*), native to Africa, belongs to Cichlidae of the Perciformes. As an important and the third largest cultured fish in the world, tilapia has been widely cultivated in tropical and subtropical areas. As a warm-water fish, tilapia has a growth temperature range of 16–38 °C and a suitable temperature range of 26–29 °C, with poor low-temperature tolerance (Wohlfarth & Hulata, 1981). Its sensitivity to low-temperature environments often leads to slow growth and even mass death. Low temperature can affect tilapia’s metabolism, immune function, cell cycle and other life activities (Dellagostin et al., 2022; Velmurugan, Chan & Weng, 2019; Yilmaz et al., 2021). When the temperature is below 15 °C, the body of tilapia will be damaged, and the semi-lethal low temperature is about 10 °C (Cnaani, Gall & Hulata, 2000; Li et al., 2002; Tave, 1990). In recent years, extreme weather has occurred more frequently, which has a significant impact on the tilapia aquaculture industry. As a result, a large amount of effort has been required to ensure the safe overwintering of tilapia. Its poor low-temperature tolerance has always been one of the bottlenecks restricting the development of the tilapia industry; how to improve the low-temperature tolerance has become one of the critical problems facing the further development of its aquaculture industry. At present, the main cultivation species of tilapia include blue tilapia (*Oreochromis aureus*), nile tilapia (*Oreochromis niloticus*), mozambique tilapia (*Oreochromis mossambicus*) and various hybrid species. The growth capacity, disease resistance and low-temperature resistance of various tilapia species significantly differ. For example, in a comparative feeding experiment on different tilapia species, *O. niloticus* was shown to possess superior growth rates and feed conversion rate (Day, Salie & Stander, 2016). In addition, tilapia galilea had an innate advantage against *Streptococcus agalactiae* infection when compared with Nile tilapia NEW GIFT strain and Nile tilapia JA strain (Huang et al., 2013). The semi-lethal low temperature of *O. aureus* was 7.82–8.44 °C, and that of *O. niloticus* was 9.39–10.13 °C (Charo-Karisa et al., 2005; Cnaani, Gall & Hulata, 2000; Nobrega et al., 2020). Therefore, it is of great significance to study the differences in the low-temperature tolerance of tilapia strains for the selection and breeding of tilapia varieties with low temperature tolerance.

Post-translational modifications (PTMs) are covalent chemical modifications of amino acids after translation, thereby changing the properties of proteins (Uversky, 2013). It is an important reason why living organisms can carry out complex physiological processes under the condition of a limited number of genes (Mann & Jensen, 2003). Phosphorylation modification is one of the most important post-translational modification methods of proteins. It is the reversible process of transferring a phosphate group on ATP or GTP to the amino acid residue of the substrate protein under the action of protein kinase, involving almost all physiological processes of the body (Cohen, 1982; Iakoucheva et al., 2004; Nirujogi et al., 2015). In eukaryotic organisms, one-third of the proteins are phosphorylated at any time (Ficarro et al., 2002). In recent years, the omics technology has been widely applied to study protein phosphorylation modification, covering various research areas, including physiology, stress resistance, drug research and environmental research (Babur et al., 2020; Huang et al., 2018; Wang et al., 2020). Therefore, the analysis of post-translational modification of tilapia’s proteome is of great potential to further understand its response mechanism to low-temperature stress.

As a protein quantitative technology, iTRAQ (isobaric tags for relative or absolute quantitation) can simultaneously analyze up to 8–10 samples of proteins, and is a commonly used high-throughput screening technology in proteomics (He et al., 2019). At present, it has been widely used in the research of phosphorylated proteome. Accordingly, this technology should be more than adequate to reveal the effect of low-temperature on protein phosphorylation modification of tilapia. As an important digestive, metabolic, detoxifying and immune organ of fish, the liver would undergo important changes when fish were exposed to low temperature stress (Chen et al., 2022; Jiao et al., 2020; Wang et al., 2022b). Hence, two tilapias (*O. aureus* and *O. niloticus*) with different low-temperature resistance were selected as target species, and comparative study on the changes of protein phosphorylation modification in the liver of these two species under low-temperature stress was performed. The results could provide basic information on the response mechanism of tilapia to low-temperature stress at the molecular level, and help improving the low-temperature tolerance of tilapia.

## Materials & Methods

The care, handling, and sampling of fish were performed following animal care protocols approved by the Laboratory Animal Centre of Yangtze River Fisheries Research Institute, Chinese Academy of Fishery Sciences.

### Experimental fish and experimental method

*O. aureus* and *O. niloticus* were obtained from the Freshwater Fisheries Research Center of Chinese Academy of Fisheries Sciences (Wuxi, Jiangsu, China) and transported to the Yangtze River Fisheries Research Institute (Wuhan, Hubei, China).

The initial body weight and length of *O. aureus* and *O. niloticus* were 20.09 ± 2.76 g and 19.61 ± 2.86 g, 7.69 ± 1.02 cm and 7.56 ± 0.94 cm, respectively. Before commencing experiments, the fish were domesticated for two weeks at 30 °C in an indoor recirculating aquaculture system with 20 fish per 450-L polyethylene cultivation tanks. After that, a refrigerator was used to reduce the water temperature from 30 °C to 10 °C by 1 °C per day. Fish were hand-fed to apparent satiation or cessation of feeding three times daily (08:30, 12:30 and 16:40). Normal feeding was maintained during the cooling period and the low-temperature stress experiment. The dissolved oxygen was detected daily. The other water quality parameters were monitored in the morning once a week. The water pH was 7.2−7.5, the dissolved oxygen was higher than 5.0 mg/L, and the total ammonia nitrogen was lower than 0.5 mg/L during the culture phase. The formulation of the diet in this experiment was show in Table S1.

### Sample preparation

After the tilapias were respectively kept at 30 °C and 10 °C for 24 h, liver samples were collected. A total of sixteen *O. niloticus* were collected at 10 and 30 °C, anesthetized with tricaine methane sulfonate solution (100 mg/L) (MS-222, GREENHX Biological Technology Co. Ltd., Beijing, China). And then the fish were dissected on ice, the liver was quickly isolated and placed in a two mL cryopreservation tube at −80 °C for proteomic analysis. More specifically, the liver of eight *O. niloticus* collected from four tanks (two fish per tank) were sampled at 30 °C, and each four liver samples were mixed to form two single pools for the iTRAQ experiment. Similarly, another two liver sample pools were also collected at 10 °C. *O. aureus* samples were collected in the same way as for *O. niloticus*.

### Protein extraction and trypsin digestion

The liver sample was ground into powder in liquid nitrogen and transferred to a centrifuge tube. The ice-cold lysis buffer (7 M urea, 2 M thiourea, 4% SDS, 40 mM Tris–HCl, pH 8.5 1 mM PMSF, and 2 mM EDTA) was added to liver samples and maintained at 4 °C for 5 min; then, DTT was added to reach a final concentration of 10 mM. The lysed samples were centrifuged at 15,000×g for 30 min at 4 °C to remove cell debris after being sonicated for 10 min on ice. The supernatant was precipitated by adding four times volumes of cold acetone. The precipitated protein was resuspended in 8 M urea/100 mM TEAB (pH 8.0). The proteins were then reduced with 10 mM dithiothreitol at 56 °C, alkylated with 50 mM iodoacetamide in the dark and diluted four times with 100 mM TEAB (pH 8.0) prior to overnight digestion with trypsin (1:50 w/w). The resulting peptides were desalted with Sep-Pak C18 column (Waters, MA, USA).

### iTRAQ labeling and phosphopeptides enrichment

The experimental procedure of labeling peptides with iTRAQ and phosphopeptides enrichment followed that of Chang et al. (2018).

### LC-MS/MS analysis

All samples were analyzed using a Q Exactive HF-X mass spectrometer (Thermo Fisher Scientific, Waltham, MA, USA) coupled with the UltiMate 3000 RSLC nanosystem (Thermo Fisher Scientific, Waltham, MA, USA). Peptides were injected onto a C18 trap column (3 µm, 120 Å, 100 µm × 20 mm) and eluted at 300 nL/min onto a C18 analytical column (2 µm, 120 Å, 750 µm × 150 mm). The eluted peptides were sprayed into the mass spectrometer and scanned in the data-dependent acquisition (DDA) mode. The full MS settings were as follows: resolution: 60,000; scan range: 350–1800 m/z; AGC: 3e^6^; maximum injection time: 20 ms. The MS/MS settings were: resolution: 15,000; AGC: 2e^5^; maximum injection time: 100 ms.

### Protein identification and bioinformatics analysis

The raw data were processed and the iTRAQ-labeled proteome was quantitatively analyzed by Maxquant software (1.6.17.0). The retrieval database was from National Center for Biotechnology Information (GCF_001858045.2 for *O. niloticus* and GCF_013358895.1 for *O. aureus*). The quantitative method was set to iTRAQ 8plex and the FDR (false discovery rate) was adjusted to < 0.01. The reverse decoy database was employed to identifications. Trypsin/P was specified as the cleavage enzyme. Oxidation on Met, N-terminal acetylation of protein, desamidization of asparagine and phosphorylation of serine, threonine and tyrosine were specified as variable modifications. Domain analysis was performed using Interproscan 5.0 (IPR). Gene Ontology (GO) annotations of proteomic and phosphoproteomic data were performed based on the UniProt-GOA database (http://www.ebi.ac.uk/GOA/). The Kyoto Encyclopedia of Genes and Genomes (KEGG) database (https://www.genome.jp/kegg/) was used for annotation analysis of protein pathways, and protein interaction analysis was performed using STRING 11.5.

### Statistical analysis

Difference analysis was performed using the in-house *t*-test. The *p*-value ≤ 0.05, and the fold change ≥1.5 (up-regulated expression) or the fold change ≤0.67 (down-regulated expression) were considered significant changes. All data were subjected to a *t*-test using SPSS 19.0 (Michigan Avenue, Chicago, IL, USA). Enrichment analysis is calculated using hypergeometric tests, with *p-* value ≤ 0.05 as the threshold.

## Results

### Quality analysis of mass spectrometry data

#### Peptide length distribution and protein coverage distribution

**Figure 1:**
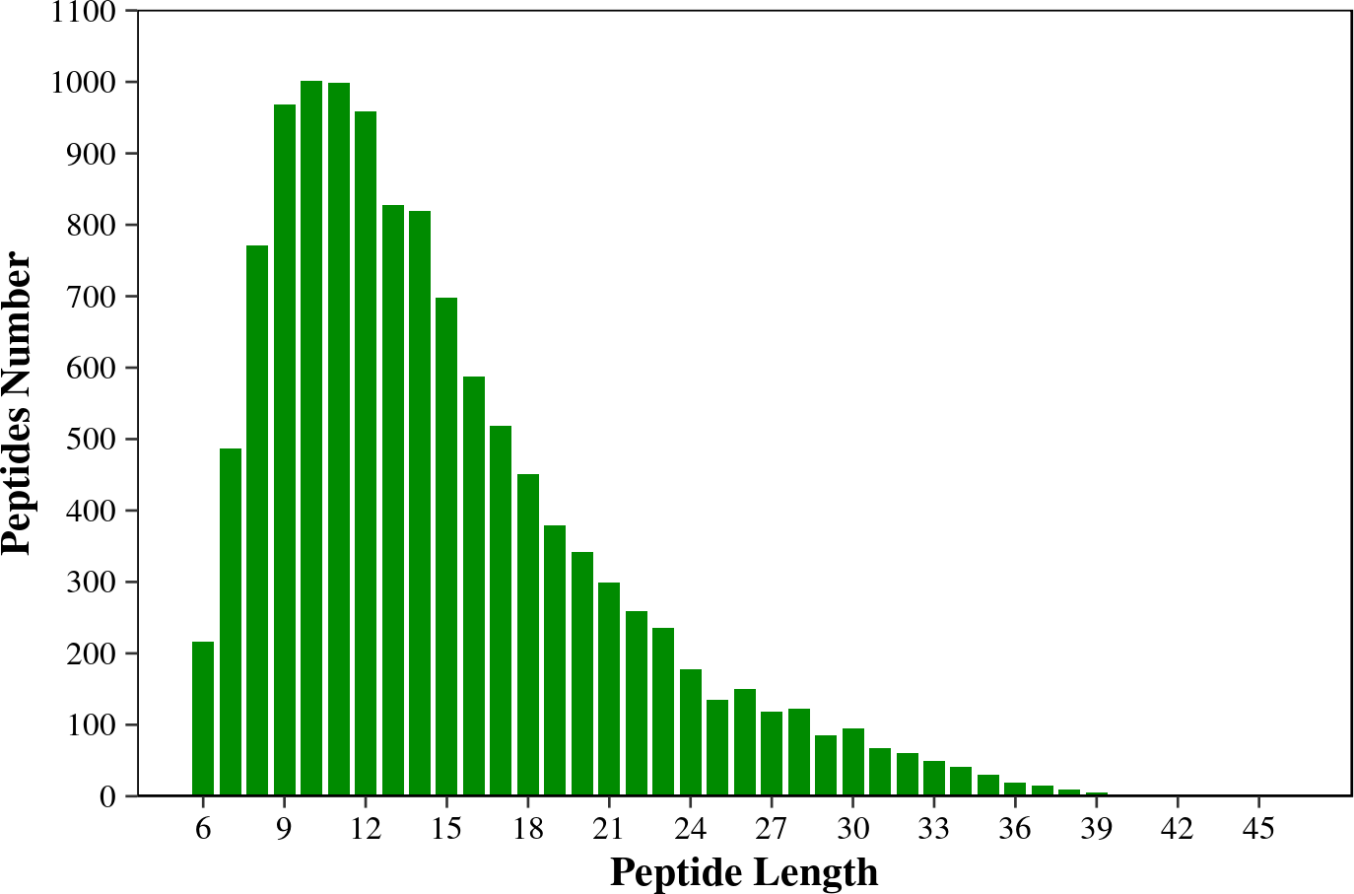
The length distribution of the total identified peptides from two tilapias. The horizontal axis shows the length of the peptides (*i.e.*, the number of amino acids), and the vertical axis shows the number of peptides.

**Figure 2:**
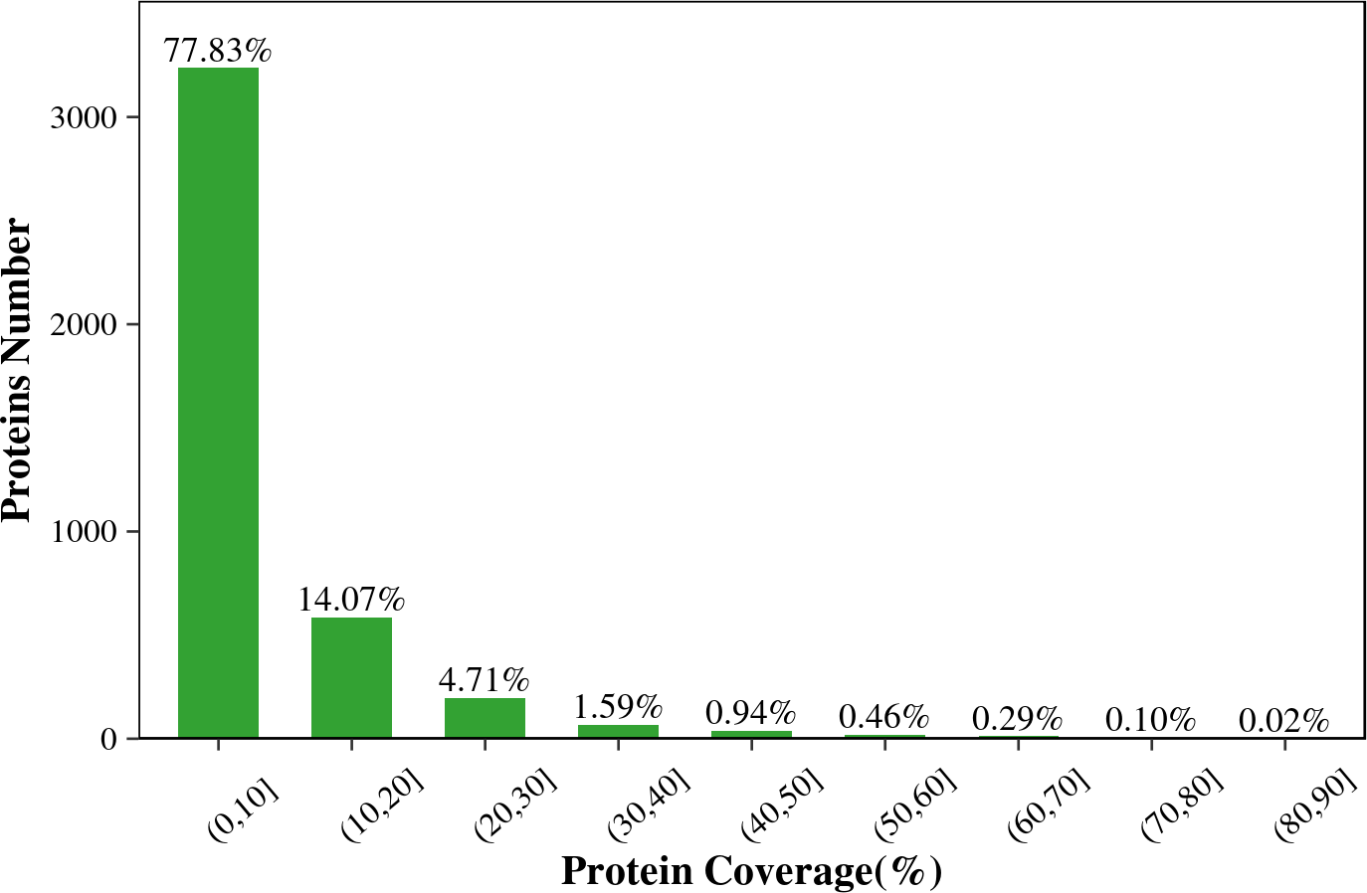
The protein coverage distribution of the total identified proteins in the two tilapias.

Figure 1 shows that the length of the identified peptides mainly distributed between seven and 17 amino acids, which was consistent with the molecular weight range detected by the mass spectrometer. Among that, the number of the identified peptides with 9-13 amino acids exceeds 900. As shown in Fig. 2, the coverage ranges for the identified proteins are concentrated in (0-10) and (10-20). The rate of number of (0-10) protein coverage exceeds 70%.

### Generation and analysis of the proteome and phosphoproteomics dataset

The data obtained by mass spectrometry were filtered for validity. For *O. niloticus*, a total of 11,333 peptides and 3,890 proteins were obtained from liver tissue, with 9,573 modification sites on 3,184 modified proteins identified. For *O. aureus* liver tissue, a total of 11,478 peptides, 3,891 proteins, and 9,706 modification sites on 3,197 modified proteins were identified (Table 1).

### Analysis of protein phosphorylation modification sites

**Figure 3:**
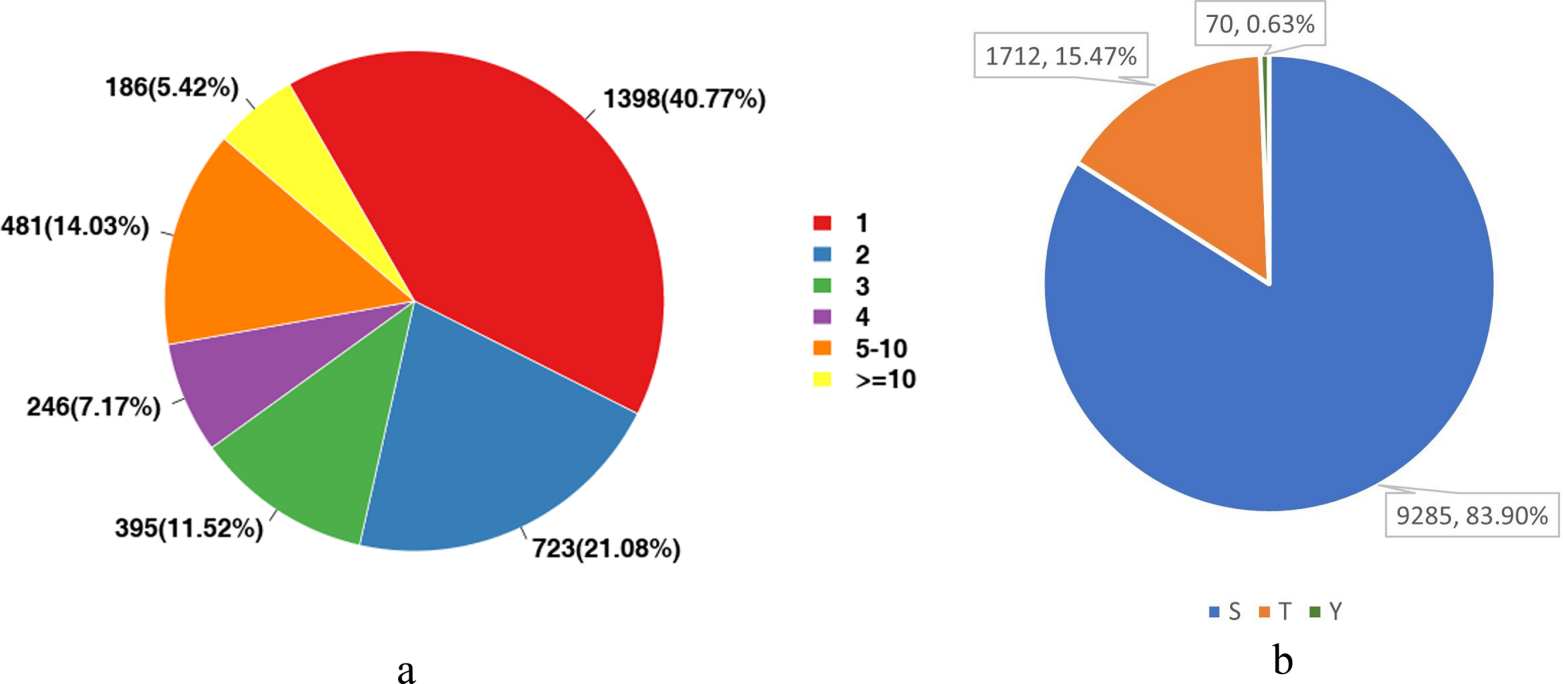
Statistical analysis of protein modification sites. (A) The distribution of the number of phosphorylation sites on phosphorylated proteins; (B) The proportional distribution of phosphorylation sites on Ser (S), Thr (T) and Tyr (Y).

We analyzed the number of phosphorylation sites of all identified proteins in the liver tissues. As shown in Fig. 3A, proteins with one-site phosphorylation dominated all identified ones, accounting for about 40.77% of all identified proteins, followed by those phosphorylated at two sites (21.08%) and five sites (14.03%).

For eukaryotes, phosphorylation modifications occur primarily at serine (S), threonine (T) and tyrosine (Y) sites. Therefore, we also counted the proportion of these three sites among all detected sites. Figure 3B shows that the main phosphorylation site was serine (S), which accounted for 83.9% of all phosphorylation sites.

### Global identification of proteomics and phosphoproteomic alterations in *O. aureus* and *O. niloticus*

The proteomic analysis on *O. aureus* liver shows that 3,891 proteins were identified and 1019 quantified in total (Table 2). For *O. niloticus* liver, 1,026 out of 3,890 identified proteins were quantified (Table 2).

For *O. niloticus* at 10 °C and 30 °C, comparative proteomics shows that 832 phosphorylation sites corresponding to 645 phosphoproteins were significantly differentially expressed, including 456 up-regulated expressed phosphosites (corresponding to 337 phosphoproteins) and 376 down-regulated expressed phosphosites (corresponding to 308 phosphoproteins) (Table 3). A total of 104 (13.2%) phosphoproteins with different expressed phosphosites were present in the proteome expression dataset. Moreover, the phosphorylation sites and protein expression levels of five phosphorylated proteins (myoglobin, ATP-citrate synthase, coatomer subunit alpha, NADP-dependent malic enzyme and uncharacterized protein) were significantly decreased.

**Table 1:** Generation of the proteome and phosphoproteomics dataset from two tilapias.

**Species**	**Id PSMs**	**Peptides**	**Proteins**	**PTM sites**	**PTM proteins**
*O. niloticus*	23275	11333	3890	9573	3184
*O. aureus*	23753	11478	3891	9706	3197

**Table 2:** Proteomics results for the liver from *O. niloticus* and *O. aureus*.

**Species**	**Proteins quantified**	**Proteins quantified**
*O. niloticus*	3891	1019
*O. aureus*	3890	1026

**Table 3:** Global statistics of phosphoproteomic in *O. niloticus* and *O. aureus*.

**Species**	Down-regulated phosphosites (corresponding to phosphoproteins)	Up-regulated phosphosites (corresponding to phosphoproteins)	Phosphoproteins present in the proteome expression datase
*O. niloticus*	376 (308)	456 (337)	104
*O. aureus*	255 (209)	231 (172)	52

In the case of *O. aureus*, 486 phosphorylation sites corresponding to 381 phosphoproteins were significantly differentially expressed between 10 °C and 30 °C groups, including 231 up-regulated (corresponding to 172 phosphoproteins) and 255 down-regulated expressed phosphosites (corresponding to 209 phosphoproteins) (Table 3). Comparison between proteomics and phosphoproteomics findings shows 52 (12.1%) phosphoproteins with different expressed phosphosites in the proteome expression dataset. In addition, one phosphorylated protein (NADP-dependent malic enzyme) was found to decrease in the phosphorylation site and the protein expression level.

### Functional enrichment analysis of phosphoproteomics

We performed functional enrichment analyses of GO, KEGG, and IPR for the phosphorylated proteins with significantly differential expressed phosphorylation sites.

#### GO enrichment analysis

**Figure 4:**
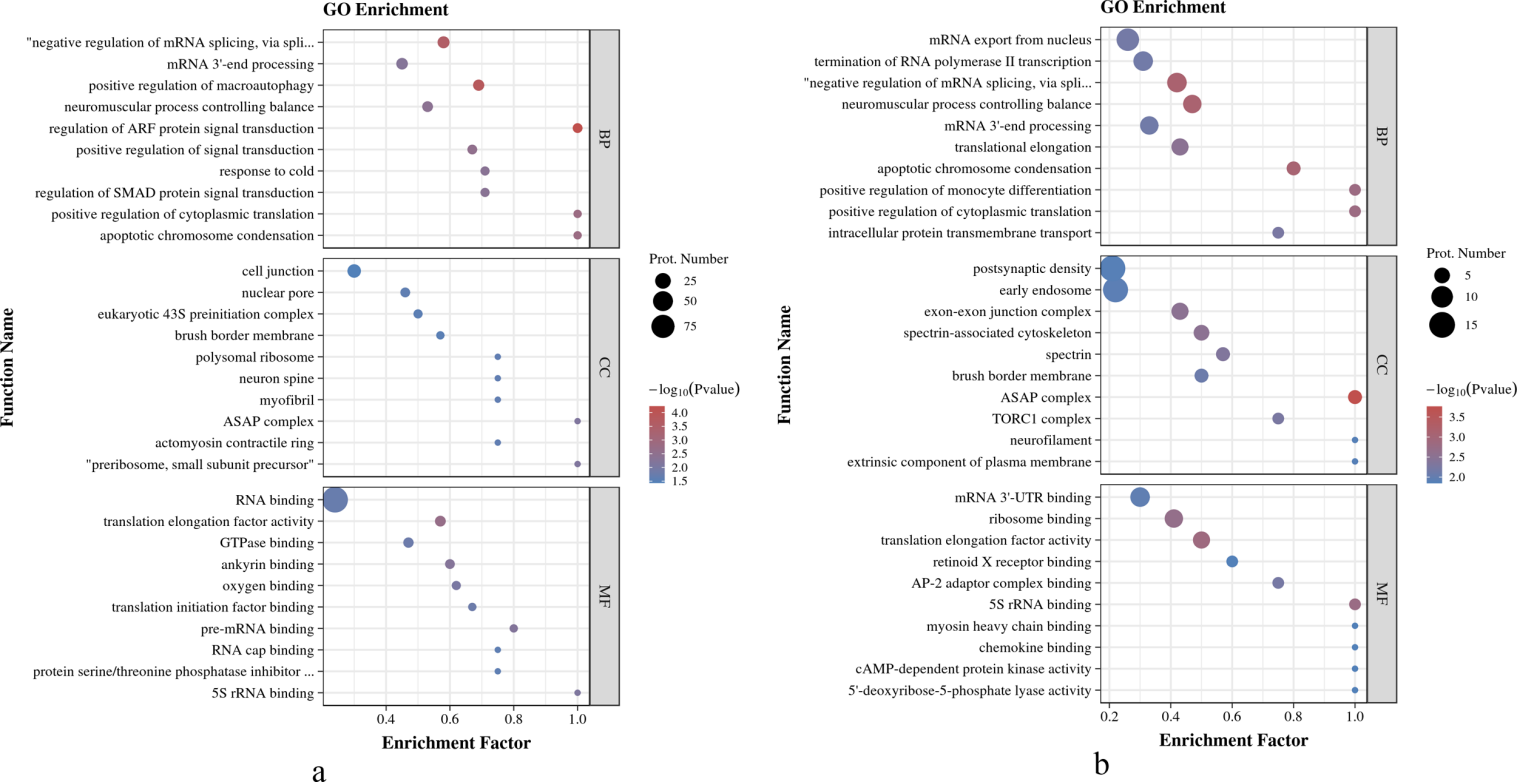
GO enrichment analysis of phosphorylated proteins with significantly differential phosphorylation sites from *O. niloticus* and *O. aureus*. (A) GO enrichment analysis of phosphorylated proteins from *O. niloticus*; (B) GO enrichment analysis of phosphorylated proteins from *O. aureus*.

As shown in Fig. 4A, in *O. niloticus*, phosphorylated proteins were enriched in the top five biological process (BP) terms, including regulation of ARF protein signal transduction, positive regulation of macroautophagy, negative regulation of mRNA splicing, *via* spliceosome, apoptotic chromosome condensation and positive regulation of cytoplasmic translation; enriched in the top five molecular function (MF) terms: translation elongation factor activity, ankyrin binding, pre-mRNA binding, 5S rRNA binding and oxygen binding; enriched in the top three cellular component (CC) terms: preribosome, small subunit precursor, ASAP complex and myofibril. As shown in Fig. 4B, in *O. aureus*, phosphorylated proteins were enriched in the top five BP terms, including negative regulation of mRNA splicing, *via* spliceosome, neuromuscular process controlling balance, apoptotic chromosome condensation, positive regulation of cytoplasmic translation and positive regulation of monocyte differentiation; enriched in the top five MF terms: translation elongation factor activity, 5S rRNA binding, ribosome binding, AP-2 adaptor complex binding and mRNA 3′-UTR binding; and enriched in the first three CC terms: ASAP complex, exon-exon junction complex and spectrin-associated cytoskeleton.

#### KEGG enrichment analysis

**Figure 5:**
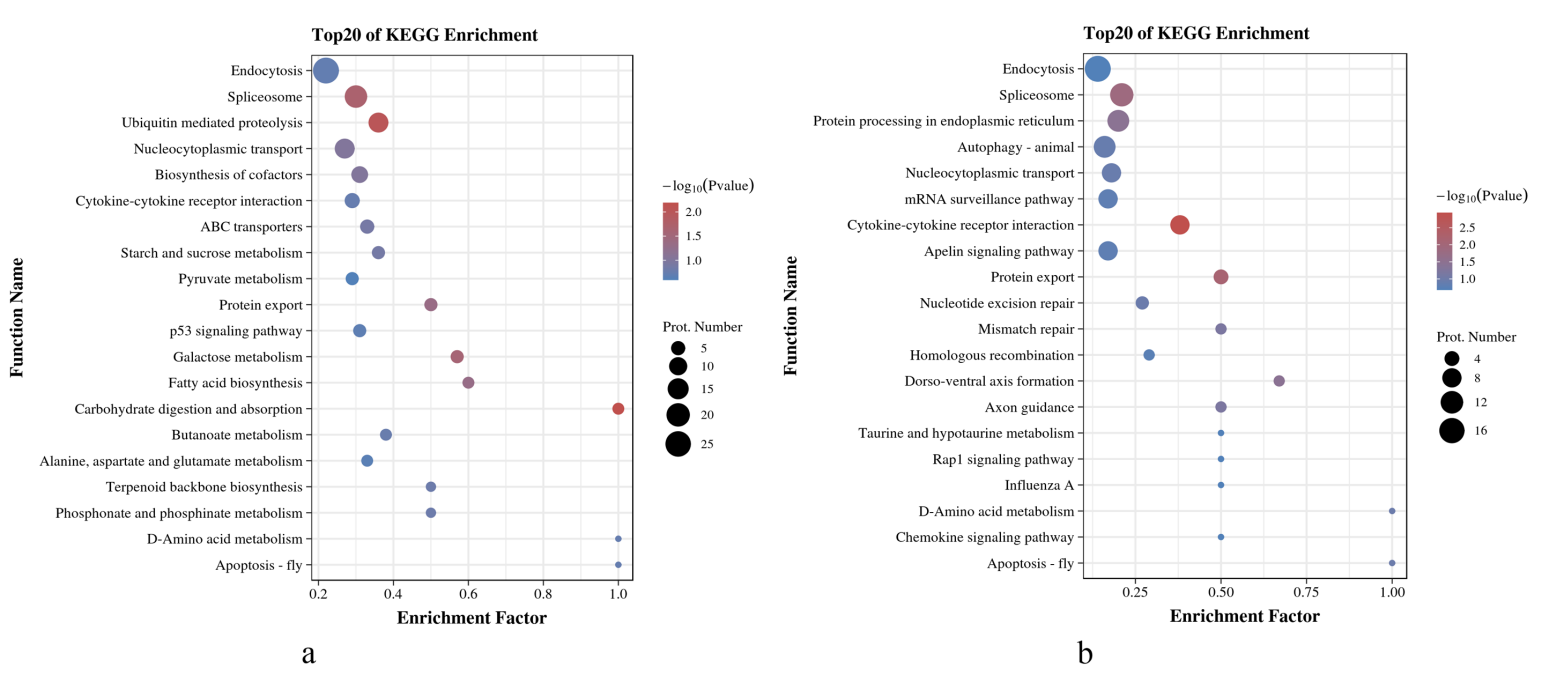
KEGG enrichment analysis of phosphorylated proteins with significantly differential phosphorylation sites from *O. niloticus* and *O. aureus*. (A) KEGG enrichment analysis of phosphorylated proteins from *O. niloticus*; (B) KEGG enrichment analysis of phosphorylated proteins from *O. aureus*.

As shown in Figs. 5A and 5B, in *O. niloticus*, phosphorylated proteins corresponding to differential expressed phosphorylation sites were mainly enriched in carbohydrate digestion and absorption, ubiquitin-mediated proteolysis, spliceosome and other pathways. As for *O. aureus*, the phosphorylated proteins were mainly enriched in cytokine-cytokine receptor interaction, protein export, spliceosome and other pathways.

#### IPR enrichment analysis

**Figure 6:**
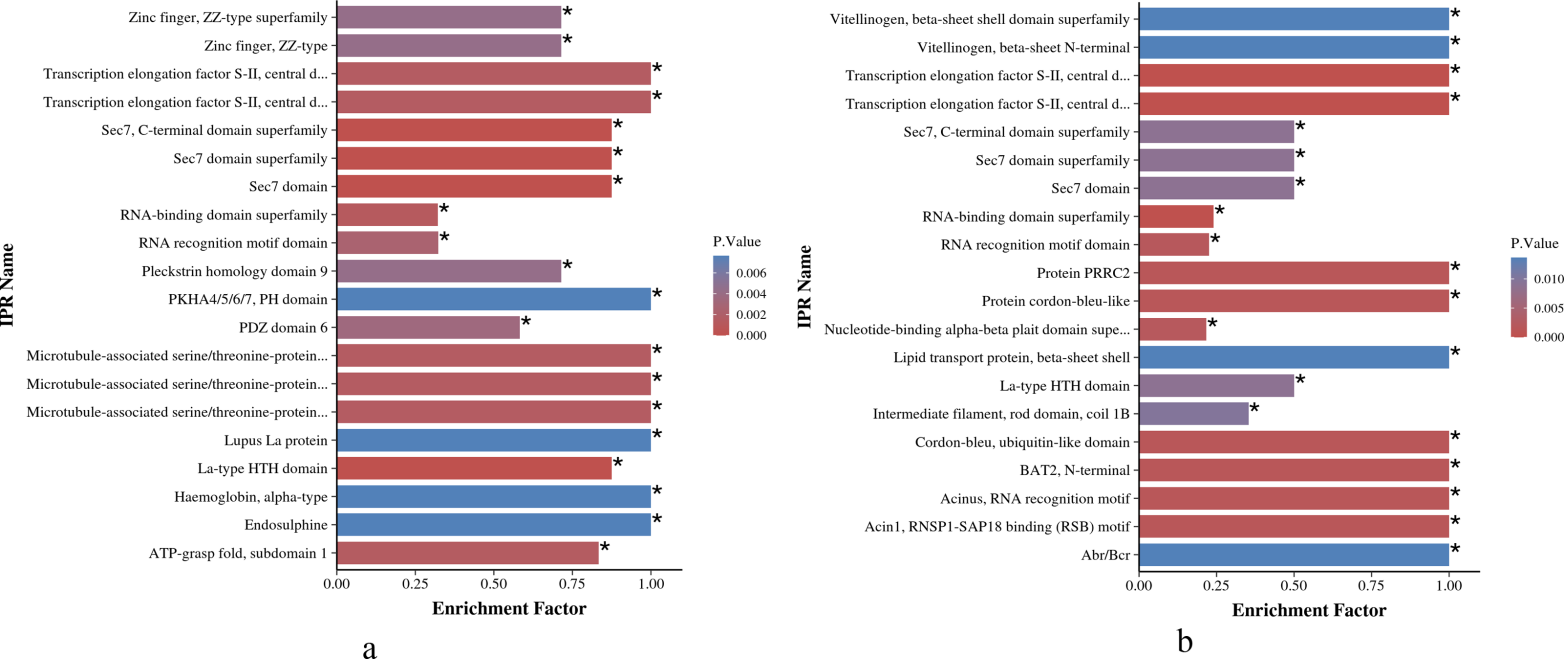
IPR enrichment analysis of phosphorylated proteins with significantly differential phosphorylation sites from *O. niloticus* and *O. aureus*. (A) IPR enrichment analysis of phosphorylated proteins from *O. niloticus*; (B) IPR enrichment analysis of phosphorylated proteins from *O. aureus*. IPRs with a P value ≤0.05 are marked with an asterisk (*).

As shown in Figs. 6A and 6B, the IPR enrichment of phosphorylated proteins corresponding to differential expressed phosphorylation sites showed certain similarities between *O. niloticus* and *O. aureus*. Transcription elongation factor S-II, central domain superfamily, transcription elongation factor S-II, Sec7 domain, RNA-binding domain superfamily and other terms were highly enriched in both species. However, the levels of enrichment significance of these domains were inconsistent between the two tilapia species. In addition, there are also many enrichment differences between these two tilapias. For example, more proteins with zinc finger domain enrich in *O. niloticus*, and in *O. aureus* includes acinus motif and so on.

### Interaction analysis of phosphorylated proteins

**Figure 7:**
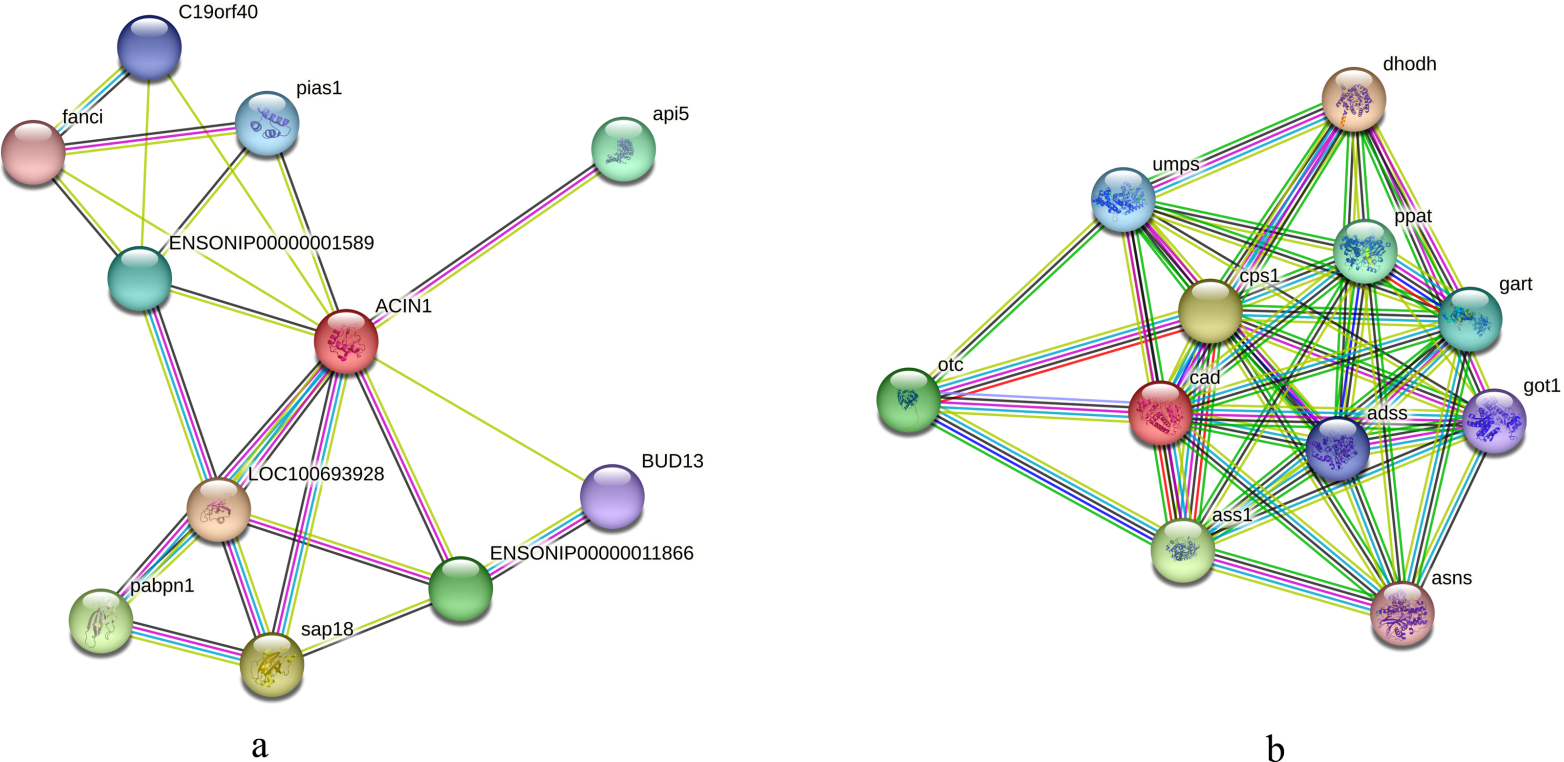
Interaction analysis of differentially phosphorylated proteins. (A) Interaction analysis of the apoptotic chromatin condensation inducer in the nucleus based on the reference library of *O. niloticus*; (B) Interaction analysis of CAD protein based on the reference database of zebrafish.

We initially compared the enrichment of differentially expressed phosphorylated proteins between the two tilapias and analyzed the first few terms with highest enrichment significances. Under low-temperature stress, the apoptotic chromatin condensation inducer and CAD protein, which were enriched in negative regulation of mRNA splicing term and apoptotic chromosome condensation term, showed similar differences in phosphorylation reduction levels. In this regard, we conducted the interaction analysis for these proteins. Analysis based on the *O. niloticus* reference library revealed that, the proteins that may interact with the apoptotic chromatin condensation inducer in the nucleus included Api5, Sap18, Pabpn and some undefined proteins, etc. Based on the zebrafish reference library, however, proteins such as Otc, Cps1 and Ass1 may interact with CAD protein (Figs. 7A, 7B).

**Figure 8:**
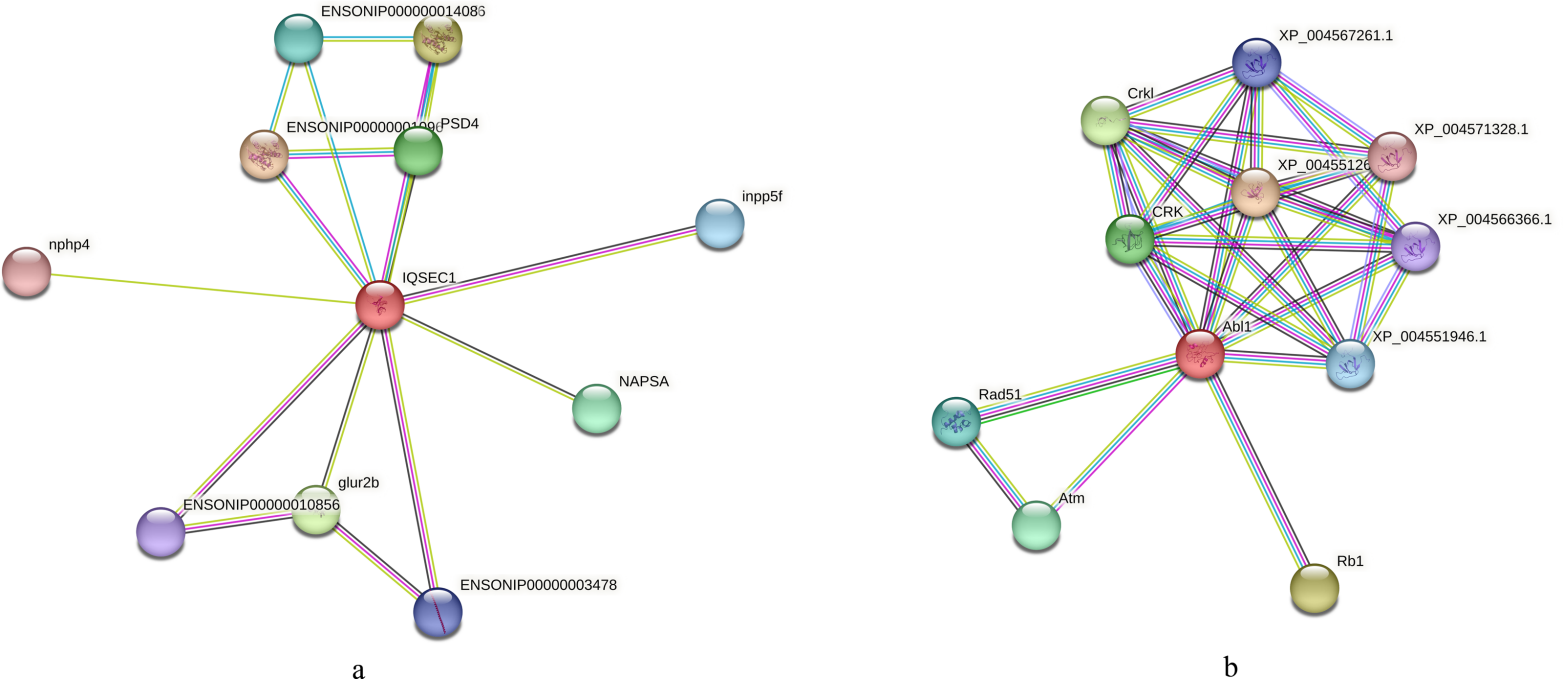
Interaction analysis of differentially phosphorylated proteins. (A) Interaction analysis of IQ motif and SEC7 domain-containing proteins based on the reference library of *O. niloticus*; (B) Interaction analysis of tyrosine-protein kinase ABL1 based on the reference database of zebrafish.

We also conducted an interaction analysis on the IQ motif and SEC7 domain-containing proteins in the regulation of ARF protein signal transduction term in both species, which differed more significantly in *O. niloticus*. In *O. aureus*, the neuromuscular process controlling balance and positive regulation of monocyte differentiation terms enriched phosphorylated proteins, such as tyrosine-protein kinase ABL1. Based on the *O. niloticus* database, the proteins that may interact with IQ motif and SEC7 domain-containing proteins included Inpp5f, Psd4, Glur2b, etc. Using the zebrafish database as a reference, the proteins that may interact with tyrosine-protein kinase ABL1 included Rad51, CRK, ATM, etc. Figs. 8A, 8B.

## Discussion

Post-translational modification is an essential way by which eukaryotes regulate the biological function of proteins. Phosphorylation is one of the most important post-translational modifications of protein, and it is a reversible process. This reversible process involves almost all physiological processes of the body, including cell signal transduction, gene expression, cell proliferation, development, differentiation and apoptosis, nerve activity, tumorigenesis, and so on (Johnson, 2009; Nirujogi et al., 2015; Weber, Schreiber & Daub, 2012).

In this study, we found that protein expression of NADP-dependent malic enzyme changed significantly in the livers of two tilapias, and its phosphorylation level also showed significant changes. The NADP-dependent malic enzyme is mainly involved in the physiological process of lipid metabolism (Barroso et al., 2001; Chang & Tong, 2003; Hsieh et al., 2019). Studies have confirmed that the dynamic regulation of phosphorylation and acetylation of malic enzyme affects lipid metabolism, and NADP-dependent malic enzyme becomes inactive after phosphorylation (Zhu et al., 2020). In addition, low temperatures can increase malic enzyme synthesis, thus increasing the synthesis of fatty acids. The newly synthesized fatty acids are mainly used in membrane phospholipids synthesis, thus resisting low-temperature stress (Viola et al., 1988). When fish are subjected to low-temperature stress, lipid metabolism has been reported to be one of the earliest biological processes that are significantly altered (Abdel-Ghany et al., 2021; Nobrega et al., 2020; Snyder & Hennessey, 2003). In this study, the significant changes in protein expression and phosphorylation level of NADP-dependent malic enzyme in the two tilapias also suggested the vital role of lipids as a critical energy source in fish’s response to low-temperature stress. Besides, the lipid levels might be correlated with the low-temperature tolerance of fish. Moreover, many studies also found that the expression of many genes related to lipid metabolism changed significantly when fish were under low-temperature stress (Liu et al., 2020; Nie et al., 2016; Sun et al., 2019). In this study, the basic biological process of the two tilapias when coping with the low temperature is similar.

In addition, by comparing the enrichment of differentially phosphorylated proteins in the livers of the two tilapias and analyzing the top terms with high enrichment significance, we found that a variety of phosphorylated proteins were involved in similar biological processes in the two tilapias facing low-temperature stress. For example, many proteins involved in the related apoptotic biological process were significantly enriched. Many studies have demonstrated that apoptosis is involved in the response of fish to low-temperature stress (Hu et al., 2016; Reid et al., 2022; Ren et al., 2021). When fish were subjected to low-temperature stress, the various apoptosis pathways, namely the mitochondrial pathway, endoplasmic reticulum pathway and death receptor pathway, had significant changes. For instance, the expression of many apoptosis-related genes, such as the caspase family and the BCL-2 family, was up-regulated in orange-spotted grouper at cold temperatures (Sun et al., 2019). When zebrafish are exposed to the low temperature, cold can induce endoplasmic reticulum (ER) stress and apoptosis (Chen et al., 2019). In addition, in our previous studies, many apoptosis-related genes, including GAS2 genes and BCL-2 family genes, participated in the process of tilapia coping with low-temperature stress (Yang et al., 2017; Yang et al., 2020).

In this study, we found that the phosphorylation levels of apoptotic chromatin condensation inducer in the nucleus (ACINl) and CAD proteins changed significantly in both tilapias (see Table S2). ACINl is an RNA-binding protein directly involved in RNA processing, and it plays an important role in cell apoptosis and apoptosis chromosome concentration (Lin et al., 2020; Sahara et al., 1999). In addition, the expression of ACINl may be related to fat synthesis (Lin et al., 2020). The function, stability and activity of ACINl depend on its phosphorylation (Nandi & Kramer, 2018). However, considering the results of IPR enrichment analysis, the protein with acinus motif was more significantly enriched in *O. aureus*. It is speculated that those difference in phosphorylation protein in the two tilapias may affect their low temperature tolerance. CAD protein is a multifunctional enzyme with DNase activity. Caspases can cleave CAD inhibitors and activate CAD, which causes DNA cleavage and cell apoptosis (Enari et al., 1998; Kimura et al., 2004; Larsen et al., 2010). The activity of CAD protein is regulated by protein phosphorylation reaction (Nakashima et al., 2013). In this study, we also analyzed the protein interaction network of ACINl and CAD. We found that the proteins that may interact with these two proteins are involved in physiological activities such as transcriptional, post-translational regulation and apoptosis. For example, the Api5 (apoptosis inhibitor 5) participates in cell apoptosis as an effective inhibitor (Chen et al., 2021), whereas the Sap18 and Pabpn are involved in the post-transcriptional and post-translational modification processes, respectively (Nicholson & Pasquinelli, 2019; Zhang et al., 1997).

At the same time, we further compared the different expressed phosphorylated proteins that were enriched in the apoptotic chromosome condensation in the two tilapias and found notable differences between the two tilapias. For example, the phosphorylation level of Serine/Arginine-Rich Splicing Factor 6 (SRS F6) was significantly up-regulated in *O. niloticus*, while that of Serine/Arginine-Rich Splicing Factor 9 (SRS F9) was significantly down-regulated in *O. niloticus*. But those was just the opposite in *O. aureus* (see Table S2). The serine/arginine-rich splicing factors (SRSFs) are a class of serine/arginine-rich splicing factors, *i.e.,* a class of trans-acting factors that play a crucial role in pre-mRNA splicing (Wagner & Frye, 2021). As members of the SRSF family, SRSF6 and SRSF9 can promote cell proliferation and migration (Jensen, Wilkinson & Krainer, 2014; Wang et al., 2022). Meanwhile, SRSF6 has also been found to be involved in the alternative splicing of apoptosis-related *FAS* gene (Choi et al., 2022). SRSF9 can inhibit caspases 3/7 and reduce cell apoptosis. The phosphorylation of SRSF6 and SRSF9 regulates the function of their alternative splicing (Li et al., 2021; Ninomiya et al., 2020). However, there is still much to learn about the function of SRSF. In this study, the phosphorylation differences of SRSF between the two tilapias under low-temperature stress may be associated with their different cell apoptosis levels, but its function needs further analysis.

Additionally, phosphorylated proteins involved in the regulation of ARF protein signal transduction and positive regulation of macroautophagy showed significant changes in *O. niloticus*. These proteins included several ARF-related proteins, such as the IQ motif and sec7 domain-containing protein, as well as the brefeldin a-inhibited guanine nucleotide-exchange protein 2 (see Table S2). ARF protein signal transduction has multiple physiological functions, including secretion, endocytosis, phagocytosis and signal transduction (Donaldson & Jackson, 2011). The activity of ARF protein is mainly regulated by binding GTP (Goldberg, 1998). However, in *O. aureus*, the top five significantly enriched BP terms of differentially phosphorylated proteins included the neuromuscular process controlling balance and positive regulation of monocyte differentiation, which was different from that in *O. niloticus*. The proteins involved in these BP terms included SH3, multiple ankyrin repeat domains protein 3 and tyrosine-protein kinase ABL1 (see Table S2). The function of these proteins is mainly related to nerve development, cell division and growth (Kim et al., 2020; Luan et al., 2021). Together, our results indicated that the physiological processes involved in the low temperature tolerance acted differently in the two tilapias.

According to the IPR enrichment analysis in the two tilapias, although the differentially phosphorylated proteins were enriched to many similar domains, for example, the SEC7 domain, the enriched significance of phosphorylated proteins with the same domain in *O. aureus* was less than that in *O. niloticus*. In these domains, the SEC7 domain is a conservative core domain in Arf family proteins (Mouratou et al., 2005; Pipaliya et al., 2019), that is responsible for the catalytic function that catalyzes the exchange of GDP for GTP on the ARF family of small GTP-binding proteins (Donaldson & Jackson, 2000; Jackson & Casanova, 2000). ARF proteins are significant regulators of membrane dynamics and protein transport within the eukaryotic cell (D’Souza-Schorey & Chavrier, 2006). Many studies have reported that the cell membrane also plays an important role in fish subjected to low-temperature stress, and the low environmental temperature will affect the fluidity and integrity of fish cell membranes (Wang et al., 2014). The membrane of fish adapted to cold environments is more fluid than that of fish adapted to warm environments (Farkas et al., 2001). Similar to the above results, the phosphorylation level of Arf family members related to cell membrane integrity and transport changed significantly when the two tilapias were subjected to low-temperature stress in the present study. Furthermore, the differences in the enrichment level of these protein members may also be one of the factors affecting their abilities to cope with low-temperature stress.

Comparing the MF enrichment between the two tilapias, proteins corresponding to different phosphorylation sites were enriched in similar MF terms, involving the translation elongation factor activity and various binding terms. However, for the binding term, there were some differences between the binding objects and the significant level of enrichment between the two tilapias. For example, ankyrin binding, mainly involved in link cytoskeleton (Gil et al., 2003), and oxygen and GTPase binding involved in energy metabolism, were more significantly enriched in *O. niloticus*. In *O. aureus*, the AP-2 adaptor complex binding of clathrin-mediated endocytosis from the plasma membrane (Holman & Sandoval, 2001), mRNA 3′-UTR binding and retinoid X receptor binding were more significantly enriched, which play an important role in post-transcriptional regulation (Son & Paton, 2020).

In addition, the results of the KEGG enrichment analysis showed some differences between the two tilapias. For example, the carbohydrate digestion and absorption pathways were more enriched in *O. niloticus*. The oxidative decomposition of carbohydrates is an essential energy source for fish. Many studies have confirmed that low temperature significantly reduces the initial energy reserve (including lipids and carbohydrates) of fish and significantly increases energy consumption (Wen et al., 2017). At the same time, the expressions of genes related to energy metabolism pathway also change significantly (Qian & Xue, 2016; Xu et al., 2018). Our results indicate that low-temperature stress had a more severe effect on the energy metabolism of *O. niloticus*. The more prominent pathways in *O. aureus* were the cybernetic-cybernetic receptor interaction, protein export and so on. Further analysis, this pathway contains interferon alpha/beta receptor 2, interleukin-1 receptor, C-X-C chemokine receptor, and growth hormone receptor genes. It indicating that the immunity and growth of *O. aureus* may be more affected by low-temperature stress.

## Conclusions

To summarize, the comparative phosphorylation proteomics showed that the main physiological activities (*e.g.*, energy metabolism, immunity, apoptosis, *etc.*) affected by low-temperature stress in the two tilapias was similar, and the difference in endurance of low temperature of these two tilapias might be related to the differences in categories, expression and modification level of genetic products which were involved in the aforementioned physiological processes.

##  Supplemental Information

10.7717/peerj.15599/supp-1 Supplemental Information 1 The formulation and proximate composition of the experimental diet

10.7717/peerj.15599/supp-2 Supplemental Information 2 GO enrichment from *O. niloticus* and *O. aureus*

10.7717/peerj.15599/supp-3 Supplemental Information 3 O. niloticus 30vs10 Diff expressed report

10.7717/peerj.15599/supp-4 Supplemental Information 4 O. aureus 30vs10 Diff expressed report

10.7717/peerj.15599/supp-5 Supplemental Information 5 Raw data of 30vs10 in O. niloticus

10.7717/peerj.15599/supp-6 Supplemental Information 6 Raw data of 30vs10 in O. aureus

10.7717/peerj.15599/supp-7 Supplemental Information 7 ARRIVE 2.0 Checklist

## Additional Information and Declarations

### DNA Deposition

The following information was supplied regarding the deposition of DNA sequences: The data of peptide sequencing is available in the Supplemental Files.

### Data Availability

The following information was supplied regarding data availability: The raw measurements are available in the Supplementary Files.

## References

[ref-1] Abdel-Ghany HM, Salem ME, Ezzat AA, Essa MA, Helal AM, Ismail RF, El-Sayed AM (2021). Effects of different levels of dietary lipids on growth performance, liver histology and cold tolerance of Nile tilapia (*Oreochromis niloticus*). Journal of Thermal Biology.

[ref-2] Babur O, Melrose AR, Cunliffe JM, Klimek J, Pang J, Sepp AI, Zilberman-Rudenko J, Tassi Yunga S, Zheng T, Parra-Izquierdo I, Minnier J, McCarty OJT, Demir E, Reddy AP, Wilmarth PA, David LL, Aslan JE (2020). Phosphoproteomic quantitation and causal analysis reveal pathways in GPVI/ITAM-mediated platelet activation programs. Blood.

[ref-3] Barroso JB, Peragon J, Garcia-Salguero L, De la Higuera M, Lupianez JA (2001). Carbohydrate deprivation reduces NADPH-production in fish liver but not in adipose tissue. International Journal of Biochemistry and Cell Biology.

[ref-4] Chang GG, Tong L (2003). Structure and function of malic enzymes, a new class of oxidative decarboxylases. Biochemistry.

[ref-5] Chang J, Tian J, Zhu Y, Zhong R, Zhai K, Li J, Ke J, Han Q, Lou J, Chen W, Zhu B, Shen N, Zhang Y, Gong Y, Yang Y, Zou D, Peng X, Zhang Z, Zhang X, Huang K, Yang M, Wang L, Wu C, Lin D, Miao X (2018). Exome-wide analysis identifies three low-frequency missense variants associated with pancreatic cancer risk in Chinese populations. Nature Communications.

[ref-6] Charo-Karisa H, Rezk MA, Bovenhuis H, Komen H (2005). Heritability of cold tolerance in Nile tilapia, Oreochromis niloticus, juveniles. Aquaculture.

[ref-7] Chen H, Zhao F, Chen K, Guo Y, Liang Y, Zhao H, Chen S (2022). Exposure of zebrafish to a cold environment triggered cellular autophagy in zebrafish liver. Journal of Fish Diseases.

[ref-8] Chen K, Li X, Song G, Zhou T, Long Y, Li Q, Zhong S, Cui Z (2019). Deficiency in the membrane protein Tmbim3a/Grinaa initiates cold-induced ER stress and cell death by activating an intrinsic apoptotic pathway in zebrafish. Journal of Biological Chemistry.

[ref-9] Chen M, Wu W, Liu D, Lv Y, Deng H, Gao S, Gu Y, Huang M, Guo X, Liu B, Zhao B, Pang Q (2021). Evolution and structure of API5 and its roles in anti-apoptosis. Protein and Peptide Letters.

[ref-10] Choi N, Jang HN, Oh J, Ha J, Park H, Zheng X, Lee S, Shen H (2022). SRSF6 regulates the alternative splicing of the apoptotic fas gene by targeting a novel RNA sequence. Cancers.

[ref-11] Cnaani A, Gall G, Hulata G (2000). Cold tolerance of tilapia species and hybrids. Aquaculture International.

[ref-12] Cohen P (1982). The role of protein phosphorylation in neural and hormonal control of cellular activity. Nature.

[ref-13] Day SB, Salie K, Stander HBJAI (2016). A growth comparison among three commercial tilapia species in a biofloc system. Aquaculture International.

[ref-14] Dellagostin EN, Martins AWS, Blodorn EB, RS TL, Komninou ER, Varela Junior AS, Corcini CD, Nunes LS, Remiao MH, Collares GL, Domingues WB, Giongo JL, Vaucher RA, Campos VF (2022). Chronic cold exposure modulates genes related to feeding and immune system in Nile tilapia (*Oreochromis niloticus*). Fish & Shellfish Immunology.

[ref-15] Donaldson JG, Jackson CL (2000). Regulators and effectors of the ARF GTPases. Current Opinion in Cell Biology.

[ref-16] Donaldson JG, Jackson CL (2011). ARF family G proteins and their regulators: roles in membrane transport, development and disease. Nature Reviews Molecular Cell Biology.

[ref-17] D’Souza-Schorey C, Chavrier P (2006). ARF proteins: roles in membrane traffic and beyond. Nature Reviews Molecular Cell Biology.

[ref-18] Enari M, Sakahira H, Yokoyama H, Okawa K, Iwamatsu A, Nagata S (1998). A caspase-activated DNase that degrades DNA during apoptosis, and its inhibitor ICAD. Nature.

[ref-19] Farkas T, Fodor E, Kitajka K, Halver JE (2001). Response of fish membranes to environmental temperature. Aquaculture Research.

[ref-20] Ficarro SB, McCleland ML, Stukenberg PT, Burke DJ, Ross MM, Shabanowitz J, Hunt DF, White FM (2002). Phosphoproteome analysis by mass spectrometry and its application to *Saccharomyces cerevisiae*. Nature Biotechnology.

[ref-21] Gil OD, Sakurai T, Bradley AE, Fink MY, Cassella MR, Kuo JA, Felsenfeld DP (2003). Ankyrin binding mediates L1CAM interactions with static components of the cytoskeleton and inhibits retrograde movement of L1CAM on the cell surface. Journal of Cell Biology.

[ref-22] Goldberg J (1998). Structural basis for activation of ARF GTPase: mechanisms of guanine nucleotide exchange and GTP-myristoyl switching. Cell.

[ref-23] He C, Kong L, Puthiyakunnon S, Wei H-X, Zhou L-J, Peng H-J (2019). iTRAQ-based phosphoproteomic analysis reveals host cell’s specific responses to *Toxoplasma gondii* at the phases of invasion and prior to egress. Biochimica Et Biophysica Acta (BBA)—Proteins and Proteomics.

[ref-24] Holman GD, Sandoval IV (2001). Moving the insulin-regulated glucose transporter GLUT4 into and out of storage. Trends in Cell Biology.

[ref-25] Hsieh JY, Shih WT, Kuo YH, Liu GY, Hung HC (2019). Functional roles of metabolic intermediates in regulating the human mitochondrial NAD(P)(+)-dependent malic enzyme. Scientific Reports.

[ref-26] Hu P, Liu M, Liu Y, Wang J, Zhang D, Niu H, Jiang S, Wang J, Zhang D, Han B, Xu Q, Chen L (2016). Transcriptome comparison reveals a genetic network regulating the lower temperature limit in fish. Scientific Reports.

[ref-27] Huang J, Wu Z, Wang J, Zhang X (2018). Quantitative phosphoproteomics reveals GTBP-1 regulating C.elegans lifespan at different environmental temperatures. Biochemical and Biophysical Research Communications.

[ref-28] Huang BF, Zou LL, Xie JG, Huang ZC, Li YW, Li AX (2013). Immune responses of different species of tilapia infected with *Streptococcus agalactiae*. Journal of Fish Diseases.

[ref-29] Iakoucheva LM, Radivojac P, Brown CJ, O’Connor TR, Sikes JG, Obradovic Z, Dunker AK (2004). The importance of intrinsic disorder for protein phosphorylation. Nucleic Acids Research.

[ref-30] Jackson CL, Casanova JE (2000). Turning on ARF: the Sec7 family of guanine-nucleotide-exchange factors. Trends in Cell Biology.

[ref-31] Jensen MA, Wilkinson JE, Krainer AR (2014). Splicing factor SRSF6 promotes hyperplasia of sensitized skin. Nature Structural & Molecular Biology.

[ref-32] Jiao S, Nie M, Song H, Xu D, You F (2020). Physiological responses to cold and starvation stresses in the liver of yellow drum (*Nibea albiflora*) revealed by LC-MS metabolomics. Science of the Total Environment.

[ref-33] Johnson LN (2009). The regulation of protein phosphorylation. Biochemical Society Transactions.

[ref-34] Kim S, Kim H, Park D, Kim J, Hong J, Kim JS, Jung H, Kim D, Cheong E, Ko J, Um JW (2020). Loss of IQSEC3 Disrupts GABAergic synapse maintenance and decreases somatostatin expression in the hippocampus. Cell Reports.

[ref-35] Kimura Y, Sugimoto C, Matsukawa S, Sunaga H, Igawa H, Yamamoto H, Ito T, Saito H, Fujieda S (2004). Combined treatment of cisplatin and overexpression of caspase-activated deoxyribonuclease (CAD) promotes apoptosis in vitro and in vivo. Oral Oncology.

[ref-36] Larsen BD, Rampalli S, Burns LE, Brunette S, Dilworth FJ, Megeney LA (2010). Caspase 3/caspase-activated DNase promote cell differentiation by inducing DNA strand breaks. Proceedings of the National Academy of Sciences of the United States of America.

[ref-37] Li S, Li C, Dey M, Gagalac F, Dunham R (2002). Cold tolerance of three strains of Nile Tilapia, *Oreochromis niloticus*, in China. Aquaculture.

[ref-38] Li Y, Xu J, Lu Y, Bian H, Yang L, Wu H, Zhang X, Zhang B, Xiong M, Chang Y, Tang J, Yang F, Zhao L, Li J, Gao X, Xia M, Tan M, Li J (2021). DRAK2 aggravates nonalcoholic fatty liver disease progression through SRSF6-associated RNA alternative splicing. Cell Metabolism.

[ref-39] Lin YC, Lu YH, Lee YC, Hung CS, Lin JC (2020). Altered expressions and splicing profiles of Acin1 transcripts differentially modulate brown adipogenesis through an alternative splicing mechanism. Biochimica Et Biophysica Acta-Gene Regulatory Mechanisms.

[ref-40] Liu L, Zhang R, Wang X, Zhu H, Tian Z (2020). Transcriptome analysis reveals molecular mechanisms responsive to acute cold stress in the tropical stenothermal fish tiger barb (*Puntius tetrazona*). BMC Genomics.

[ref-41] Luan Y, Li C, Zuo W, Hu H, Gao R, Zhang B, Tong X, Lu C, Dai F (2021). Gene mapping reveals the association between tyrosine protein kinase Abl1 and the silk yield of Bombyx mori. Animal Genetics.

[ref-42] Mann M, Jensen ON (2003). Proteomic analysis of post-translational modifications. Nature Biotechnology.

[ref-43] Mouratou B, Biou V, Joubert A, Cohen J, Shields DJ, Geldner N, Jurgens G, Melancon P, Cherfils J (2005). The domain architecture of large guanine nucleotide exchange factors for the small GTP-binding protein Arf. BMC Genomics.

[ref-44] Nakashima A, Kawanishi I, Eguchi S, Yu EH, Eguchi S, Oshiro N, Yoshino K, Kikkawa U, Yonezawa K (2013). Association of CAD, a multifunctional protein involved in pyrimidine synthesis, with mLST8, a component of the mTOR complexes. Journal of Biomedical Science.

[ref-45] Nandi N, Kramer H (2018). Cdk5-mediated Acn/Acinus phosphorylation regulates basal autophagy independently of metabolic stress. Autophagy.

[ref-46] Nicholson AL, Pasquinelli AE (2019). Tales of detailed poly(A) tails. Trends in Cell Biology.

[ref-47] Nie H, Jiang L, Huo Z, Liu L, Yang F, Yan X (2016). Transcriptomic responses to low temperature stress in the Manila clam, *Ruditapes philippinarum*. Fish & Shellfish Immunology.

[ref-48] Ninomiya K, Adachi S, Natsume T, Iwakiri J, Terai G, Asai K, Hirose T (2020). LncRNA-dependent nuclear stress bodies promote intron retention through SR protein phosphorylation. The EMBO Journal.

[ref-49] Nirujogi RS, Wright Jr JD, Manda SS, Zhong J, Na CH, Meyerhoff J, Benton B, Jabbour R, Willis K, Kim MS, Pandey A, Sekowski JW (2015). Phosphoproteomic analysis reveals compensatory effects in the piriform cortex of VX nerve agent exposed rats. Proteomics.

[ref-50] Nobrega R, Banze J, Oliveira Batista R, Fracalossi D (2020). Improving winter production of Nile tilapia: What can be done?. Aquaculture Reports.

[ref-51] Pipaliya SV, Schlacht A, Klinger CM, Kahn RA, Dacks J (2019). Ancient complement and lineage-specific evolution of the Sec7 ARF GEF proteins in eukaryotes. Molecular Biology of the Cell.

[ref-52] Qian B, Xue L (2016). Liver transcriptome sequencing and de novo annotation of the large yellow croaker (*Larimichthy crocea*) under heat and cold stress. Marine Genomics.

[ref-53] Reid CH, Patrick PH, Rytwinski T, Taylor JJ, Willmore WG, Reesor B, Cooke SJ (2022). An updated review of cold shock and cold stress in fish. Journal of Fish Biology.

[ref-54] Ren J, Long Y, Liu R, Song G, Li Q, Cui Z (2021). Characterization of biological pathways regulating acute cold resistance of zebrafish. International Journal of Molecular Sciences.

[ref-55] Sahara S, Aoto M, Eguchi Y, Imamoto N, Yoneda Y, Tsujimoto Y (1999). Acinus is a caspase-3-activated protein required for apoptotic chromatin condensation. Nature.

[ref-56] Snyder R, Hennessey T (2003). Cold tolerance and homeoviscous adaptation in freshwater Alewives (*Alosa pseudoharengus*). Fish Physiology and Biochemistry.

[ref-57] Son Y, Paton CM, Ntambi JM (2020). Chapter 18—lipid metabolic features of skeletal muscle in pathological and physiological conditions. Lipid Signaling and Metabolism.

[ref-58] Sun Z, Tan X, Liu Q, Ye H, Zou C, Xu M, Zhang Y, Ye C (2019). Physiological, immune responses and liver lipid metabolism of orange-spotted grouper (*Epinephelus coioides*) under cold stress. Aquaculture.

[ref-59] Tave D (1990). Cold tolerance in tilapia. Aquaculture Magazine.

[ref-60] Uversky VN, Maloy S, Hughes K (2013). Posttranslational modification. Brenner’s Encyclopedia of Genetics.

[ref-61] Velmurugan BK, Chan CR, Weng CF (2019). Innate-immune responses of tilapia (*Oreochromis mossambicus*) exposure to acute cold stress. Journal of Cellular Physiology.

[ref-62] Viola S, Mokady S, Behar D, Cogan U (1988). Effects of polyunsaturated fatty acids in feeds of tilapia and carp: 1. Body composition and fatty acid profiles at different environmental temperatures. Aquaculture.

[ref-63] Wagner RE, Frye M (2021). Noncanonical functions of the serine-arginine-rich splicing factor (SR) family of proteins in development and disease. Bioessays.

[ref-64] Wang Q, Tan X, Jiao S, You F, Zhang PJ (2014). Analyzing cold tolerance mechanism in transgenic zebrafish (*Danio rerio*). PLOS ONE.

[ref-65] Wang X, Lu X, Wang P, Chen Q, Xiong L, Tang M, Hong C, Lin X, Shi K, Liang L, Lin J (2022). SRSF9 promotes colorectal cancer progression via stabilizing DSN1 mRNA in an m6A-related manner. Journal of Translational Medicine.

[ref-66] Wang X, Sun S, Cao X, Gao J (2020). Quantitative phosphoproteomic analysis reveals the regulatory networks of Elovl6 on lipid and glucose metabolism in zebrafish. International Journal of Molecular Sciences.

[ref-67] Wang Z, Dong Z, Yang Y, Wang J, Yang T, Chen X, Liang L, Mu W (2022b). Histology, physiology, and glucose and lipid metabolism of *Lateolabrax maculatus* under low temperature stress. Journal of Thermal Biology.

[ref-68] Weber C, Schreiber TB, Daub H (2012). Dual phosphoproteomics and chemical proteomics analysis of erlotinib and gefitinib interference in acute myeloid leukemia cells. Journal of Proteomics.

[ref-69] Wen B, Jin S-R, Chen Z-Z, Gao J-Z, Wang L, Liu Y, Liu H-P (2017). Plasticity of energy reserves and metabolic performance of discus fish (*Symphysodon aequifasciatus*) exposed to low-temperature stress. Aquaculture.

[ref-70] Wohlfarth GW, Hulata GI (1981). Applied genetics of tilapias. ICLARM Studies and Reviews.

[ref-71] Xu D, You Q, Chi C, Luo S, Song H, Lou B, Takeuchi Y (2018). Transcriptional response to low temperature in the yellow drum (*Nibea albiflora*) and identification of genes related to cold stress. Comparative Biochemistry and Physiology Part D: Genomics and Proteomics.

[ref-72] Yang C, Wang M, Wen H, Jiang M, Tian J, Lu X (2020). Genome-wide identification and expression analysis of Bcl-2 gene family under low-temperature stress in tilapia (*Oreochromis niloticus*). Israeli Journal of Aquaculture—Bamidgeh.

[ref-73] Yang C, Wu F, Lu X, Jiang M, Liu W, Yu L, Tian J, Wen H (2017). Growth arrest specific gene 2 in tilapia (*Oreochromis niloticus*): molecular characterization and functional analysis under low-temperature stress. BMC Molecular Biology.

[ref-74] Yilmaz S, Ergün S, Çelik EŞ, Banni M, Ahmadifar E, Dawood MAJJoTB (2021). The impact of acute cold water stress on blood parameters, mortality rate and stress-related genes in *Oreochromis niloticus*, *Oreochromis mossambicus* and their hybrids. Journal of Thermal Biology.

[ref-75] Zhang Y, Iratni R, Erdjument-Bromage H, Tempst P, Reinberg D (1997). Histone deacetylases and SAP18, a novel polypeptide, are components of a human Sin3 complex. Cell.

[ref-76] Zhu Y, Gu L, Lin X, Liu C, Lu B, Cui K, Zhou F, Zhao Q, Prochownik EV, Fan C, Li Y (2020). Dynamic regulation of ME1 phosphorylation and acetylation affects lipid metabolism and colorectal tumorigenesis. Molecular Cell.

