# Supplementary material for: Comparative analysis of quantitative phosphoproteomics between two tilapias (Oreochromis niloticus and Oreochromis aureus) under low-temperature stress
Source: PeerJ. 2023 Jul 10;11:e15599. doi: 10.7717/peerj.15599 (PMC10340112; doi:10.7717/peerj.15599)
Supplement: Supplemental Information 1 [file peerj-11-15599-s001.docx]

| **Ingredients** | **g/kg** | **Proximate composition** | **g/kg** |
| --- | --- | --- | --- |
| Corn gluten meal | 100 | Protein | 323 |
| Soybean meal | 220 | Lipid | 57 |
| Cottonseed meal | 80 | Ash | 77 |
| Rapeseed meal | 280 | Moisture | 103 |
| Whole wheat flour | 200 |  |  |
| Corn oil | 15 |  |  |
| Soybean oil | 15 |  |  |
| Choline chloride | 1 |  |  |
| Vitamin premix^a^ | 10 |  |  |
| Mineral premix^b^ | 40 |  |  |
| Dl-methionine | 2 |  |  |
| Bentonite | 20 |  |  |
| Sodium methoxycellulose | 10 |  |  |
| Microcrystalline cellulose | 7 |  |  |
| Total | 1000 |  |  |

a The premix provided the following per kg of diets: retinol acetate 5000 IU; cholecalciferol 2000 IU; α-tocopheryl acetate 60 mg; L-ascorbyl-2-monophosphate-Mg 120 mg; menadione 5 mg; thiamine hydrochloride 5 mg; riboflavin 20 mg; pyridoxine hydrochloride 10 mg; nicotinic acid 120 mg; calcium pantothenate 10 mg; folic acid 1 mg; biotin 0.1 mg; inositol 400 mg.

b The premix provided the following per kg of diet: Ca(H_2_PO4)_2_ 26000 mg, Ca(CH_3_CHOHCOO)_2_ 6540 mg, FeSO_4_ 42.5 mg, MgSO_4_ 1340 mg, NaH_2_PO_4_ 1744 mg, NaCl 870 mg, AlCl_3_ 3 mg, KIO_3_ 2.5 mg, KCl 1500 mg, CuCl_2_ 20 mg, MnSO_4_ 16 mg, CoCl_2_ 2 mg, ZnSO_4_ 60 mg.
