# Supplementary material for: Comparative analysis of quantitative phosphoproteomics between two tilapias (Oreochromis niloticus and Oreochromis aureus) under low-temperature stress
Source: PeerJ. 2023 Jul 10;11:e15599. doi: 10.7717/peerj.15599 (PMC10340112; doi:10.7717/peerj.15599)
Supplement: Supplemental Information 2 [file peerj-11-15599-s002.docx]

| **species** | **GO ID** | **GO Name** | **P-value** | **Down Proteins** | **Up Proteins** |
| --- | --- | --- | --- | --- | --- |
| *O. niloticus* | GO:0032012 | regulation of ARF protein signal transduction | 5.87E-05 | XP_019218706.1 LOW QUALITY PROTEIN: brefeldin A-inhibited guanine nucleotide-exchange protein 1;XP_019214106.1 IQ motif and SEC7 domain-containing protein 1 isoform X9;XP_019205095.1 IQ motif and SEC7 domain-containing protein 1 isoform X7;XP_025753221.1 Golgi-specific brefeldin A-resistance guanine nucleotide exchange factor 1;XP_005477997.1 brefeldin A-inhibited guanine nucleotide-exchange protein 2; XP_013130089.1 IQ motif and SEC7 domain-containing protein 1 isoform X3 | XP_019205095.1 IQ motif and SEC7 domain-containing protein 1 isoform X7;XP_013130089.1 IQ motif and SEC7 domain-containing protein 1 isoform X3 |
|  | GO:0016239 | positive regulation of macroautophagy | 0.000146 | XP_025756404.1 WW domain-containing adapter protein with coiled-coil isoform X3;XP_003438323.1BCL2/adenovirus E1B 19 kDa protein-interacting protein 3;XP_005460592.1 tuberin isoform X5;XP_025766699.1 la-related protein 1 isoform X7;XP_003449017.1 transcription elongation factor SPT5 isoform X2;XP_003450290.1 WW domain-containing adapter protein with coiled-coil isoform X4; XP_003440278.1 hamartin | XP_005463852.1 sequestosome-1;XP_025766699.1 la-related protein 1 isoform X7;XP_005473140.1 serine/threonine-protein kinase ULK2 isoform X3 |
|  | GO:0048025 | negative regulation of mRNA splicing, via spliceosome | 0.000266 | XP_003450162.1 RNA-binding protein with serine-rich domain 1;XP_025764222.1 serine/arginine-rich splicing factor 9 isoform X2; XP_013120948.1 apoptotic chromatin condensation inducer in the nucleus | XP_013128024.1 serine/arginine-rich splicing factor 4 isoform X2;XP_003444830.1 nuclease-sensitive element-binding protein 1 isoform X2;XP_005478377.1 serine/arginine-rich splicing factor 6 isoform X2;XP_005455446.2 splicing factor U2AF 65 kDa subunit isoform X2;XP_005464682.2 apoptotic chromatin condensation inducer in the nucleus isoform X2;XP_019204346.1 polypyrimidine tract-binding protein 1 isoform X2;XP_013120948.1 apoptotic chromatin condensation inducer in the nucleus;XP_005474903.1 heterogeneous nuclear ribonucleoprotein L isoform X1; XP_003442973.1 transformer-2 protein homolog alpha isoform X1 |
|  | GO:0030263 | apoptotic chromosome condensation | 0.00152 | XP_013120948.1 apoptotic chromatin condensation inducer in the nucleus | XP_005461572.1 LOW QUALITY PROTEIN: CAD protein;XP_005464682.2 apoptotic chromatin condensation inducer in the nucleus isoform X2;XP_013120948.1 apoptotic chromatin condensation inducer in the nucleus; XP_005448251.1 DNA topoisomerase 2-alpha |
|  | GO:2000767 | positive regulation of cytoplasmic translation | 0.00152 | **--** | XP_013121931.1 Y-box-binding protein 2 isoform X1;XP_013121932.1 Y-box-binding protein 2 isoform X2;XP_005476210.1 elongation factor 2; XP_005478746.1 elongation factor 2 |
| *O. aureus* | GO:0048025 | negative regulation of mRNA splicing, via spliceosome | 0.000715 | XP_031606966.1 nucleophosmin 1a;XP_031612890.2 apoptotic chromatin condensation inducer in the nucleus; XP_031594013.2 serine/arginine-rich splicing factor 6-like isoform X2 | XP_031605292.1 heterogeneous nuclear ribonucleoprotein L-like isoform X2;XP_039471878.1 serine/arginine-rich splicing factor 9;XP_039465691.1 apoptotic chromatin condensation inducer 1b isoform X4 ; XP_031612890.2 apoptotic chromatin condensation inducer in the nucleus;XP_031585599.2 apoptotic chromatin condensation inducer 1b isoform X1; XP_031601872.1 RNA-binding protein with serine-rich domain 1-like isoform X2 |
|  | GO:0050885 | neuromuscular process controlling balance | 0.000743 | XP_031616192.1 solute carrier family 1 member 3a;XP_031611011.1 chloride intracellular channel protein 5b isoform X1;XP_039456969.1 SH3 and multiple ankyrin repeat domains protein 3 isoform X2;XP_031598493.1 tyrosine-protein kinase ABL1 isoform X1;XP_039470876.1 SH3 and multiple ankyrin repeat domains protein 3 | XP_031591445.1 active breakpoint cluster region-related protein isoform X1;XP_031598493.1 tyrosine-protein kinase ABL1 isoform X1; XP_031598649.1 PH_BCR_vertebrate and RhoGAP_Bcr domain-containing protein isoform X1 |
|  | GO:0030263 | apoptotic chromosome condensation | 0.000807 | XP_031612890.2 apoptotic chromatin condensation inducer in the nucleus | XP_031612890.2 apoptotic chromatin condensation inducer in the nucleus;XP_039454606.1 CAD protein;XP_039465691.1 apoptotic chromatin condensation inducer 1b isoform X4; XP_031585599.2 apoptotic chromatin condensation inducer 1b isoform X1 |
|  | GO:2000767 | positive regulation of cytoplasmic translation | 0.001546 | XP_031583018.2 Y-box-binding protein 2-B isoform X1 | XP_031594175.1 elongation factor 2; XP_031582654.1 elongation factor 2-like |
|  | GO:0045657 | positive regulation of monocyte differentiation | 0.001546 | XP_031612890.2 apoptotic chromatin condensation inducer in the nucleus | XP_031585599.2 apoptotic chromatin condensation inducer 1b isoform X1;XP_039465691.1 apoptotic chromatin condensation inducer 1b isoform X4; XP_031612890.2 apoptotic chromatin condensation inducer in the nucleus |
